# AdapterRemoval: easy cleaning of next-generation sequencing reads

**DOI:** 10.1186/1756-0500-5-337

**Published:** 2012-07-02

**Authors:** Stinus Lindgreen

**Affiliations:** 1 Centre for GeoGenetics, Natural History Museum of Denmark, University of Copenhagen, Øster Voldgade 5-7, 1350 Copenhagen K, Denmark; 2 The Bioinformatics Centre, Department of Biology, University of Copenhagen, Ole Maaloes Vej 5, 2200 Copenhagen, Denmark; 3 School of Biological Sciences, University of Canterbury, Private Bag 4800, Christchurch 8041, New Zealand

**Keywords:** Next-generation sequencing, Adapter trimming, Data pre-processing, Sequence alignment, Paired-end reads, Single-end reads

## Abstract

**Background:**

With the advent of next-generation sequencing there is an increased demand for tools to pre-process and handle the vast amounts of data generated. One recurring problem is adapter contamination in the reads, i.e. the partial or complete sequencing of adapter sequences. These adapter sequences have to be removed as they can hinder correct mapping of the reads and influence SNP calling and other downstream analyses.

**Findings:**

We present a tool called AdapterRemoval which is able to pre-process both single and paired-end data. The program locates and removes adapter residues from the reads, it is able to combine paired reads if they overlap, and it can optionally trim low-quality nucleotides. Furthermore, it can look for adapter sequence in both the 5’ and 3’ ends of the reads. This is a flexible tool that can be tuned to accommodate different experimental settings and sequencing platforms producing FASTQ files. AdapterRemoval is shown to be good at trimming adapters from both single-end and paired-end data.

**Conclusions:**

AdapterRemoval is a comprehensive tool for analyzing next-generation sequencing data. It exhibits good performance both in terms of sensitivity and specificity. AdapterRemoval has already been used in various large projects and it is possible to extend it further to accommodate application-specific biases in the data.

## Findings

### Background

With the growing use of next-generation sequencing techniques in research groups all around the world there is also a growing need for tools that can help in the downstream analyses of the vast amounts of sequencing data produced. Moreover, as sequencing costs continue to drop [1], more and more groups can afford next-generation sequencing which further increases the need for efficient and accurate pre-processing of sequencing data. Consequently many groups have to deal with the same type of problems which leads to the in–house development of tools that already exist.

One of the problems encountered in many experiments — especially as read lengths keep expanding — is the sequencing of adapter fragments. If the read length, *L*_*R*_, is longer than the insert size, *L*_*I*_, then the read produced by the sequencing machine will include *L*_*A*_ = *L*_*R*_ − *L*_*I*_ nucleotides from the adapter sequence. Depending on the library building protocol used, adapter fragments will be present in the 3’ end of the read and possibly also the 5’ end. If these fragments — denoted adapter contamination in this paper — are not removed correctly they can lead to either missed alignments because the sequenced construct does not match the genome or, if the read is mapped to the genome, a misleading increase in the number of mismatches in the end of the mapping. These reads containing adapter contamination can then lead to wrong genotyping and SNP calls further downstream in the analyses. Mismatches in the 5’ end due to adapter contamination has an even higher probability of wrongfully discarding a genuine match since most mapping tools depend on a high-similarity seed region in the 5’ end of the reads (e.g. the default behaviour of Bowtie [2], BWA [3], SOAP [4], and SOAP2 [5] is to allow no more than 2 mismatches in the seed region).

The problem becomes more dramatic the shorter the molecule of interest is — for instance when sequencing microRNAs, or within the field of ancient DNA – although the problem is not isolated to these fields of research. It is therefore of great importance to clean the reads by removing these subsequences of non–genomic origin before mapping to a reference genome or performing *de novo* assembly of the reads. Since this is a general problem many different programs exist that try to solve it, each exhibiting their own strengths and weaknesses as summarized in Table 1. These methods vary in which features they offer when trimming adapters (e.g. handling single-end or paired-end data, finding adapters in the 5’ or 3’ end of reads, searching for multiple different adapters), and in what additional analyses can be performed such as trimming low-quality nucleotides or sorting the reads based on multiplexing barcodes.

**Table 1:** Comparison of various tools for trimming adapters

		**Adapter trimming**					**Quality control**		**Other**	
Method	5’	3’	SE	PE	Merge	Multi	Ns	Q	Barcode	Refs.
AdapterRemoval	Yes	Yes	Yes	Yes	Yes	No	Yes	Yes	No	[6]
Btrim	Yes	Yes	Yes	Yes	No	No	No	Yes	Yes	[7,8]
^CANGS1,2^	No	Yes	Yes	No	No	Yes	(Yes)	(Yes)	Yes	[9,10]
Cutadapt	Yes	Yes	Yes	No	No	Yes	No	Yes	No	[11,12]
EA-Tools	No	Yes	Yes	Yes	No	No	Yes	Yes	Yes	[13]
FAR^3^	Yes	Yes	Yes	Yes	No	Yes	No	Yes	Yes	[14]
FASTX^1^	No	Yes	Yes	No	No	No	(Yes)	No	Yes	[15]
Scythe	No	Yes	Yes	No	No	No	No	No	No	[16]
SeqPrep	No	Yes	No	Yes	Yes	No	No	No	No	[17]
SeqTrim	No	Yes	Yes	No	No	Yes	Yes	Yes	No	[18,19]
TagCleaner	Yes	Yes	Yes	No	No	Yes	No	No	No	[20,21]
TagDust^4^	(Yes)	Yes	Yes	(Yes)	No	Yes	No	No	No	[22,23]
Trim Galore!^3^	No	Yes	Yes	(Yes)	No	No	No	Yes	No	[24]
trimLRPatterns	Yes	Yes	Yes	No	No	No	No	No	No	[25,26]
Trimmomatic	No	Yes	Yes	Yes	No	Yes	No	Yes	No	[27]

The table summarizes the features of different programs that are aimed at removing adapter sequences from next-generation sequencing reads. For each method, the table shows if it is able to i) identify adapters in the 5’ end of reads, ii) identify adapters in the 3’ end of reads, iii) process single-end reads, iv) process paired-end reads, v) collapse overlapping pairs, vi) search for multiple different adapters, vii) trim subsequences of Ns, viii) trim low-quality nucleotides, ix) sort multiplexed reads based on barcodes. The last feature is not as such related to adapter trimming but since it occurs often in these programs it is included. The table also lists references for each program. Notes: 1) If chosen, reads with one or more Ns are discarded (i.e. Ns are not trimmed). 2) If chosen, low-quality reads are discarded (i.e. low-quality nucleotides are not trimmed). 3) Discards remaining read if the other read in a pair is removed due to trimming. 4) The aim of this program is slightly different as it compares the reads to a library of sequences and checks for significant overlap.

We present a standalone tool, AdapterRemoval, that efficiently solves most of these problems simultaneously without the need for calling multiple different programs. AdapterRemoval can find adapters in both the 5’ and 3’ end of the reads, it can handle both single-end and paired-end data, it can remove low-quality regions and trim Ns from the reads, and it can collapse overlapping paired-end reads. AdapterRemoval has been independently developed in our group where it has been used (although as an unnamed part of the pipeline) in a number of large sequencing projects mainly focused on ancient DNA [28-30]. The tool has therefore been used frequently over the years and is still an integral part of the work at the Centre of Excellence in GeoGenetics in Copenhagen. AdapterRemoval has been updated and extended based on feedback and requests from the users to solve various problems encountered. It is a versatile tool that is easy to use on any UNIX-based platform.

### Methods

**Figure 1:**
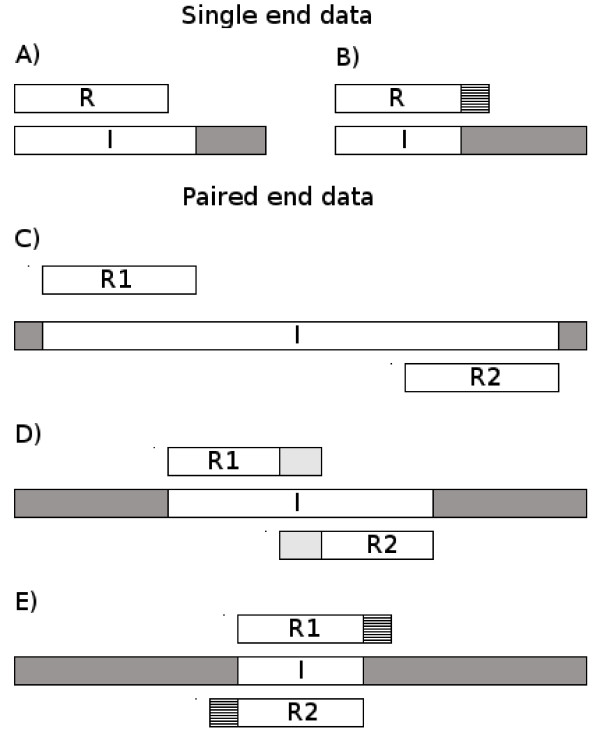
**Illustration of different constructs and the reads produced.** Single-end data on top, paired-end below. Inserts are denoted *I*, single-end reads *R* and paired-end reads *R*1 and *R*2. Read length denoted *L*_*R*_, insert length denoted *L*_*I*_. A) *L*_*I*_ ≥ *L*_*R*_: No adapter contamination. B) *L*_*I*_ < *L*_*R*_: adapter contamination occurs in 3’ end. C) *L*_*I*_ ≥ 2· *L*_*R*_: No adapter contamination and no overlap between reads. D) *L*_*R*_ < *L*_*I*_ < 2  · *L*_*R*_: No adapter contamination but the two reads overlap. E) *L*_*I*_ < *L*_*R*_: adapter contamination in 3’ ends of both reads, overlap between 5’ ends of reads. This information can be used to perform the pairwise alignment needed (after reverse complementing mate 2 from the pair) to locate adapter contamination and/or overlap between reads

AdapterRemoval uses a variation of the Needleman–Wunsch algorithm [31] that has been altered to perform ungapped semiglobal alignment looking for matches between the 3’ end of the read and the 5’ end of the adapter sequence. The specificities of the algorithm depends on the data (single- or paired-end) and on other settings as described in the following. The overall functionality of the program is illustrated in Figure 1.

If the insert being sequenced is shorter than the read length, the read will include part of the adapter sequence in the 3’ end. In case of single-end reads AdapterRemoval performs alignment between the reads and the expected adapter sequence. When processing single-end reads the identification of the adapter fragment becomes increasingly difficult the shorter it is.

When analyzing paired-end data much more information is available: If we have adapter contamination it will be symmetrical in the two reads (if no indels occur, see Figure 1E) and the program can identify precisely even a single nucleotide from the adapters. The two reads (with one being reverse-complemented) will be identical in the overlapping region (i.e. the genomic insert) and the 5’ and 3’ ends of the reads, respectively, will match the adapters. Even when allowing for mismatches this makes the procedure extremely sensitive to adapter contamination in these cases.

The allowed mismatch rate can be set by the user but by default the program demands perfect match for alignments up to 5 nucleotides, allows 1 mismatch for alignments up to 10 nucleotides, and allows a fraction (0.15 for paired-end and 0.33 for single-end) of the alignment length to be mismatches for longer alignments. The program employs a simple yet effective scoring scheme: 1 for matches, -1 for mismatches, 0 for alignments to Ns. The alignment chosen is the best one in terms of total score where the number of mismatches is in the allowed range.

**Figure 2:**
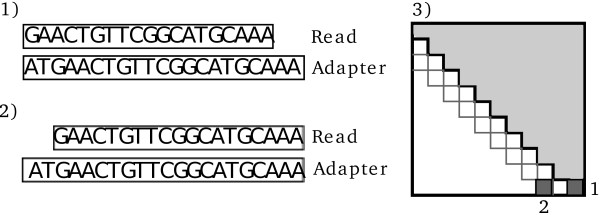
**The need for shifting the alignment due to missing nucleotides.** If the read is missing a few nucleotides in the 5’ end, the proper alignment will not be recoverable if the procedure stops at the first position. As shown in 1), this leads to multiple mismatches and possibly missed adapter contamination. If the alignment is shifted by *S* nucleotides as shown in 2), the correct alignment can be found. The dynamic programming matrix in 3) shows which entries in the matrix leads to the two solutions shown here. The light grey part is the upper half of the matrix that is calculated by default; the two dark grey entries illustrate the two alignments shown in 1) and 2)

As gaps are much less common than mismatches in Illumina data [32] we do not include gapped alignments, and since we only calculate alignments between the 3’ end of the read and the 5’ end of the adapter we only need the top half of the dynamic programming matrix above the main diagonal (Figure 2, panel 3). Observations have shown that reads sometimes miss a few bases in the 5’ end. This can lead to adapter contamination being missed as the alignment is confined to the top half of the matrix thereby not aligning the two reads properly (Figure 2, panel 1 and 2). To solve this the alignments can be extended slightly which effectively shifts one read towards the 3’ end by *S* nucleotides relative to the other read (default is *S*=2). This creates an overhang in the 5’ end of the adapter where the first few nucleotides are ignored since they are not in the sequenced read. The cost of this is that *S* additional subdiagonals have to be calculated in the matrix to include these alignments (Figure 2, panel 3).

AdapterRemoval allows overlapping pairs of reads to be collapsed into a single read whether the two contain adapter contamination or not (see Figure 1). This idea has also been pursued independently in the program FLASH published recently [33]. If the insert length, *L*_*I*_, is longer than the read length, *L*_*R*_, but shorter than 2 · *L*_*R*_, then we have no adapter contamination but the two reads overlap in their 3’ ends. In that case the two reads can be combined into one read and the qualities for the overlap can be reestimated based on the two quality strings. If the two reads in a pair contain adapter sequence the remaining overlapping fragments of genomic origin will be from the same original sequence and can likewise be collapsed into one read and the qualities re-estimated.

To collapse two reads into one, AdapterRemoval treats the quality scores for the overlapping region as a position specific scoring matrix (PSSM). For each position in the overlap, we have a nucleotide and a quality score from both reads. The quality score, Q, can be converted to an error probability, *P*_*e*_ = 10^−*Q*/10^. This gives the probability for the nucleotide in the read, *P*_1_ = 1 −*P*_*e*_, and a probability for each of the remaining three nucleotides, *P*_2_ = *P*_*e*_ /3. These probabilities are combined for the two reads to give a single re-estimated probability distribution for the overlapping region. Finally, the most likely nucleotide sequence is chosen based on the PSSM, and the probabilities are translated back into re-estimated Phred quality scores [34]. If the user decides to do so AdapterRemoval will detect these cases and output the new nucleotide sequence and re-estimated quality scores. The user can specify how long the overlap has to be for two reads to be combined (default is 11 nucleotides as in [35]).

It is well-known that the quality of a read is lower in the ends [32], with elevated error rates at both the 5’ and – in particular – 3’ end of the reads. AdapterRemoval has therefore been designed to deal with this in two different ways: It is possible to trim consecutive stretches of the ambiguity character N from both ends of the reads. Since the presence of Ns can make mapping hard due to an increased number of spurious hits it is normally important to remove these uninformative positions. Furthermore, AdapterRemoval can trim the reads based on the quality scores by removing consecutive stretches of nucleotides from both ends of the reads where the quality scores do not exceed a given threshold (default is to trim 2 or lower). These two trimming options can of course be used either alone or in combination. Related to this the program also has the option of discarding reads that contain too many Ns even after trimming. How many Ns to allow is defined by the user.

Using AdapterRemoval it is also possible to find and remove adapters from the 5’ end of the reads. However, due to the difference in the experimental setup this is done in a different and more strict manner than in the 3’ end. First, at most one mismatch is tolerated in the aligned part of the read and the adapter. Second, it is expected that the 5’ adapter sequence is present in almost full length. Hence, the trimming only allows for the adapter to have slipped a few positions corresponding to the first few nucleotides of the adapter not being present in the read. This parameter can be defined by the user but the default is up to two nucleotides, and these slipped positions do not add to the number of mismatches.

When AdapterRemoval is used it reads either one or two FASTQ files and depending on the settings the output is written to a number of files. In the single-end case, one file contains the trimmed reads, and another contains discarded reads (due to e.g. length or quality control). In the paired-end case the trimmed pairs are written to two new files that keep the ordering of the pairs intact. If one mate in a pair is discarded the remaining read is written to a singleton file in order to keep as much useful data as possible. These reads can then be treated as single-end reads. All discarded reads are written to a separate file. AdapterRemoval can work with compressed files using pipes as described in the user manual, the user can specify the quality base used (either Phred + 33 (default) or Phred + 64), and the user can specify the minimum length of a read after trimming (default is 15 nucleotides).

A simulated test set was created based on a modern paired-end dataset from *Yersinia pestis* (SRA accession SRX028780). From this dataset, 1,000,000 read pairs were extracted with each read being 75 nucleotides long. For each pair, a simulated insert length between 0 and 200 nucleotides was sampled. If the insert length was 150 nucleotides or more, the reads do not overlap and no changes were made to the data. If the insert length was between 149 and 75 nucleotides, the two reads overlap but we have no adapter contamination. In this case, a subsequence based on the insert length was taken from read 1, reverse-complemented and inserted into read 2, and then the new part of read 2 was randomly mutated based on the quality scores to simulate read errors. If the insert length was shorter than 75 nucleotides, a subsequence of read 1 was copied to read 2 as above, and furthermore adapter sequence was added to both reads from two different adapters. Finally, the new sequences were mutated based on the quality scores. This yields 1,000,000 read pairs with known adapter contamination in 373,963 cases and no adapter in the remaining 626,037 pairs.

### Test results

The performance of AdapterRemoval was tested on the simulated paired-end dataset described above and compared to another program that is able to handle both single-end and paired-end data, Trimmomatic version 0.20 [27]. Trimmomatic was run as described on the website but changing the minimum read length to 15 after trimming to make it comparable to AdapterRemoval. For single-end analysis both programs were run on just the first read from each pair. In this test the programs were only used for trimming adapters and no filtering based on Ns or low-quality nucleotides was used.

After trimming, the output from each program was analyzed and five categories of cases were recorded: 

1. How often did the program trim a read that did not contain adapter?

2. How often did the program trim only the adapter sequence?

3. How often did the program trim more than the adapter sequence?

4. How often did the program trim less than the adapter sequence?

5. How often did the program not trim anything from a read with adapter contamination?

The false positive rate is the sum of cases 1 and 3, i.e. the cases where the program trimmed nucleotides that were not from the adapter (even if the adapter sequence was also removed). The true positive rate is case 2 where only the adapter is removed. The sum of cases 4 and 5 is the false negative rate since, in both cases, the program failed to remove the full adapter sequence. The number of reads containing no adapter and not being trimmed at all is the true negative rate. From these numbers, positive predictive value, sensitivity, specificity and Matthew’s correlation coefficient were calculated for both programs:

$$\;\begin{array}{ll} \text{PPV} & =\frac{\text{TP}}{\text{TP}+\text{FP}}\\ \text{SEN} & =\frac{\text{TP}}{\text{TP}+\text{FN}}\\ \text{SPEC} & =\frac{\text{TN}}{\text{TN}+\text{FP}}\\ \text{MCC} & =\frac{\text{TP}\cdot \text{TN}-\text{FP}\cdot \text{FN}}{\sqrt{\left(\text{TP}+\text{FP})(\text{TP}+\text{FN})(\text{TN}+\text{FP})(\text{TN}+\text{FN}\right)}} \end{array}$$

The results are summarized in Table 2 together with run times and maximum memory usage as reported by the UNIX time command. AdapterRemoval runs slower than Trimmomatic but uses less memory.

**Table 2:** Performance of AdapterRemoval and Trimmomatic on simulated test set

	**AdapterRemoval**				**Trimmomatic**			
	**Paired-end**		**Single-end**		**Paired-end**		**Single-end**	
Run time (s)	139.42		36.07		39.9		20.1	
Memory (kB)	7,664		6,512		387,488		611,536	
Trimmed, no adapter	95	(0.02%)	93,909	(15.00%)	0	(0.00%)	0	(0.00%)
Trimmed exact	346,806	(92.74%)	327,925	(87.69%)	120284	(32.16%)	120,284	(32.16%)
Trimmed more	0	(0.00%)	298	(0.08%)	0	(0.00%)	0	(0.00%)
Trimmed less	0	(0.00%)	1,038	(0.28%)	0	(0.00%)	0	(0.00%)
Missed adapter	27,157	(7.26%)	44,702	(11.95%)	253679	(67.84%)	253,679	(67.84%)
PPV	1.00		0.78		1.00		1.00	
SEN	0.93		0.88		0.32		0.32	
SPEC	1.00		0.85		1.00		1.00	
MCC	0.94		0.71		0.48		0.48	

Statistics for AdapterRemoval and Trimmomatic tested on both single-end and paired-end data. The test set contains 1,000,000 read pairs of which 373,963 contain adapter fragments and the remaining 626,037 do not. The paired-end test was performed on the 1,000,000 pairs, and the single-end test was performed on the first read from each pair. The reported numbers are: Run time (seconds); max memory usage (kilobytes); number of reads containing no adapter that were trimmed; number of reads with adapter where just the adapter was removed; number of reads with adapter where more than the adapter was trimmed; number of trimmed reads with adapter where the full adapter was not removed; number of reads with adapter where no trimming was performed; positive predictive value; sensitivity; specificity; Matthew’s correlation coefficient. For “Trimmed, no adapter”, the percentage of the 626,037 reads with no adapter that were trimmed is shown. For the following four rows, the percentages are of the 373,963 read pairs with adapter, and these numbers add up to 373,963 and 100%, respectively.

Trimmomatic performs equally good on both single-end and paired-end data performing the exact same trimming. It has perfect specificity and positive predictive value at a modest sensitivity, yielding a MCC of 0.48. However, when looking at the results it is clear that in the paired-end case Trimmomatic trims fewer of the reverse reads (86,043 reads are trimmed exactly) thus missing more adapters in those cases (287,920). It is not clear why it does not trim both members of a pair the same. For this test, the best numbers were used in the calculations, and the program was run with all combinations of adapter sequences (both original and reverse-complemented) to make sure that the correct sequences were tested.

In the single-end case, AdapterRemoval shows good performance with lower specificity and positive predictive value than Trimmomatic but also a much higher sensitivity and, hence, MCC of 0.71. AdapterRemoval trims many more adapters correctly but also wrongly trims more reads without adapters.

As expected, all the measures of accuracy go up when using AdapterRemoval on paired-end data compared to single-end data. The extra information available in having two reads that align in case of adapter contamination makes AdapterRemoval much better at removing only true adapter residues from the reads. This is especially clear from the false positive rate that drops by almost a factor 1000. The MCC is increased from 0.71 to 0.94.

As mentioned above, Trimmomatic was run using the the default parameters given on the website and only changing parameters to make it directly comparable to AdapterRemoval. It is likely that Trimmomatic would perform better if the parameters were tweaked which has not been done in this experiment. However, based on this test AdapterRemoval shows good performance on all measures. A test where both programs also trimmed Ns and low-quality nucleotides showed the same overall results as above. Future work on AdapterRemoval should focus on improving the run time and including an option for trimming multiple adapters simultaneously.

## Availablity and requirements

AdapterRemoval is implemented in C++ and the source code is available under GNU GPL from Google Code, http://code.google.com/p/adapterremoval/

## Competing interests

SL has declared that no competing interests exist.

## References

[B1] Niedringhaus TP Milanova D Kerby MB Snyder MP Barron AE Landscape of next-generation sequencing technologies Anal Chem 2011 83 12 4327 4341 10.1021/ac2010857 21612267 PMC3437308

[B2] Langmead B Trapnell C Pop M Salzberg SL Ultrafast and memory-efficient alignment of short DNA sequences to the human genome Genome Biol 2009 10 3 R25 10.1186/gb-2009-10-3-r25 19261174 PMC2690996

[B3] Li H Durbin R Fast and accurate short read alignment with Burrows-Wheeler transform Bioinformatics 2009 25 1754 1760 10.1093/bioinformatics/btp324 19451168 PMC2705234

[B4] Li R Li Y Kristiansen K Wang J SOAP: short oligonucleotide alignment program Bioinformatics 2008 24 5 713 714 10.1093/bioinformatics/btn025 18227114

[B5] Li R Yu C Li Y Lam TW Yiu SM Kristiansen K Wang J SOAP2: an improved ultrafast tool for short read alignment Bioinformatics 2009 25 15 1966 1967 10.1093/bioinformatics/btp336 19497933

[B6] Lindgreen S AdapterRemoval 2012 [ http://code.google.com/p/adapterremoval/]

[B7] Kong Y Btrim: a fast, lightweight adapter and quality trimming program for next-generation sequencing technologies Genomics 2011 98 2 152 153 10.1016/j.ygeno.2011.05.009 21651976

[B8] Kong Y Btrim 2011 [ http://graphics.med.yale.edu/trim/]

[B9] Pandey RV Nolte V Schlotterer C CANGS: a user-friendly utility for processing and analyzing 454 GS-FLX data in biodiversity studies BMC Res Notes 2010 3 3 10.1186/1756-0500-3-3 20180949 PMC2830946

[B10] Pandey RV Nolte V Schlotterer C CANGS 2010 [ http://i122server.vu-wien.ac.at/CANGS1.1/] 10.1186/1756-0500-3-3 PMC2830946 20180949

[B11] Martin M Cutadapt removes adapter sequences from high-throughput sequencing reads EMBnet J 2011 17 1 10 12

[B12] Martin M Cutadapt 2011 [ http://code.google.com/p/cutadapt/]

[B13] Aronesty E ea-utils: Command-line tools for processing biological sequencing data 2011 [ http://code.google.com/p/ea-utils]

[B14] Unknown FAR [ http://sourceforge.net/projects/theflexibleadap/]

[B15] Gordon A FASTX-Toolkit [ http://hannonlab.cshl.edu/fastx_toolkit/]

[B16] Buffalo V Scythe [https://github.com/vsbuffalo/scythe]

[B17] John JS SeqPrep [https://github.com/jstjohn/SeqPrep]

[B18] Falgueras J Lara AJ Fernandez-Pozo N Canton FR Perez-Trabado G Claros MG SeqTrim: a high-throughput pipeline for pre-processing any type of sequence read BMC Bioinformatics 2010 11 38 10.1186/1471-2105-11-38 20089148 PMC2832897

[B19] Falgueras J Lara AJ Fernandez-Pozo N Canton FR Perez-Trabado G Claros MG SeqTrim 2010 [ http://www.scbi.uma.es/seqtrim] 10.1186/1471-2105-11-38 PMC2832897 20089148

[B20] Schmieder R Lim YW Rohwer F Edwards R TagCleaner: Identification and removal of tag sequences from genomic and metagenomic datasets BMC Bioinformatics 2010 11 341 10.1186/1471-2105-11-341 20573248 PMC2910026

[B21] Schmieder R TagCleaner [ http://tagcleaner.sourceforge.net/]

[B22] Lassmann T Hayashizaki Y Daub C TagDust - a program to eliminate artifacts from next generation sequencing data Bioinformatics 2009 25 21 2839 2840 10.1093/bioinformatics/btp527 19737799 PMC2781754

[B23] Lassmann T Hayashizaki Y Daub C TagDust 2009 [ http://genome.gsc.riken.jp/osc/english/software/] 10.1093/bioinformatics/btp527 PMC2781754 19737799

[B24] Krueger F Trim Galore! [ http://www.bioinformatics.babraham.ac.uk/projects/trim_galore/]

[B25] Morgan M Anders S Lawrence M Aboyoun P Pages H Gentleman R ShortRead: a bioconductor package for input, quality assessment and exploration of high-throughput sequence data Bioinformatics 2009 25 19 2607 2608 10.1093/bioinformatics/btp450 19654119 PMC2752612

[B26] Morgan M Anders S Lawrence M Aboyoun P Pages H Gentleman R ShortRead 2009 [ http://bioconductor.org/packages/release/bioc/html/ShortRead.html] 10.1093/bioinformatics/btp450 PMC2752612 19654119

[B27] Bolger A Giorgi F Trimmomatic [ http://www.usadellab.org/cms/index.php?page=trimmomatic/]

[B28] Rasmussen M Li Y Lindgreen S Pedersen JS Albrechtsen A Moltke I Metspalu M Metspalu E Kivisild T Gupta R Bertalan M Nielsen K Gilbert MT Wang Y Raghavan M Campos PF Kamp HM Wilson AS Gledhill A Tridico S Bunce M Lorenzen ED Binladen J Guo X Zhao J Zhang X Zhang H Li Z Chen M Orlando L Kristiansen K Bak M Tommerup N Bendixen C Pierre TL Gr?nnow B Meldgaard M Andreasen C Fedorova SA Osipova LP Higham TF Ramsey CB Hansen TV Nielsen FC Crawford MH Brunak S Sicheritz-Ponten T Villems R Nielsen R Krogh A Wang J Willerslev E Ancient human genome sequence of an extinct Palaeo-Eskimo Nature 2010 463 757 762 10.1038/nature08835 20148029 PMC3951495

[B29] Rasmussen M Guo X Wang Y Lohmueller KE Rasmussen S Albrechtsen A Skotte L Lindgreen S Metspalu M Jombart T Kivisild T Zhai W Eriksson A Manica A Orlando L De La Vega FM Tridico S Metspalu E Nielsen K Avila-Arcos MC Moreno-Mayar JV Muller C Dortch J Gilbert MT Lund O Wesolowska A Karmin M Weinert LA Wang B Li J Tai S Xiao F Hanihara T van Driem G Jha AR Ricaut FX de Knijff P Migliano AB Gallego Romero I Kristiansen K Lambert DM Brunak S Forster P Brinkmann B Nehlich O Bunce M Richards M Gupta R Bustamante CD Krogh A Foley RA Lahr MM Balloux F Sicheritz-Ponten T Villems R Nielsen R Wang J Willerslev E An Aboriginal Australian genome reveals separate human dispersals into Asia Science 2011 334 94 98 10.1126/science.1211177 21940856 PMC3991479

[B30] Orlando L Ginolhac A Raghavan M Vilstrup J Rasmussen M Magnussen K Steinmann KE Kapranov P Thompson JF Zazula G Froese D Moltke I Shapiro B Hofreiter M Al-Rasheid KA Gilbert MT Willerslev E True single-molecule DNA sequencing of a pleistocene horse bone Genome Res 2011 21 1705 1719 10.1101/gr.122747.111 21803858 PMC3202287

[B31] Needleman SB Wunsch CD A general method applicable to the search for similarities in the amino acid sequence of two proteins J Mol Biol 1970 48 3 443 453 10.1016/0022-2836(70)90057-4 5420325

[B32] Minoche AE Dohm JC Himmelbauer H Evaluation of genomic high-throughput sequencing data generated on Illumina HiSeq and genome analyzer systems Genome Biol 2011 12 11 R112 10.1186/gb-2011-12-11-r112 22067484 PMC3334598

[B33] Magoc̆ T Salzberg SL FLASH: fast length adjustment of short reads to improve genome assemblies Bioinformatics 2011 27 2957 2963 10.1093/bioinformatics/btr507 21903629 PMC3198573

[B34] Ewing B Green P Base-calling of automated sequencer traces using phred. II. Error probabilities Genome Res 1998 8 186 194 9521922

[B35] Reich D Green RE Kircher M Krause J Patterson N Durand EY Viola B Briggs AW Stenzel U Johnson PL Maricic T Good JM Marques-Bonet T Alkan C Fu Q Mallick S Li H Meyer M Eichler EE Stoneking M Richards M Talamo S Shunkov MV Derevianko AP Hublin JJ Kelso J Slatkin M Paabo S Genetic history of an archaic hominin group from Denisova Cave in Siberia Nature 2010 468 1053 1060 10.1038/nature09710 21179161 PMC4306417

